# Metabolic reprogramming of microglia: Effects on development, disease, and therapeutic potential

**DOI:** 10.4103/NRR.NRR-D-25-01298

**Published:** 2026-01-27

**Authors:** Haiwei Zhang, Mengmeng Jin, Peng Jiang, Ying Liu

**Affiliations:** 1 Center for Translational Science, Florida International University, Port St. Lucie, FL, USA; 2 Robert Stempel College of Public Health and Social Work, Florida International University, Miami, FL, USA; 3 Department of Cell Biology and Neuroscience, Rutgers University, New Brunswick, Piscataway, NJ, USA

**Keywords:** energy metabolism, functional states, human induced pluripotent stem cells, lipid metabolism, metabolic reprogramming, microglia, neurodegenerative disease, neuroinflammation

## Abstract

Microglia, the immune sentinels of the central nervous system, play vital roles in maintaining neural homeostasis and mediating responses to injury and disease. Their functions, including synaptic pruning to neuroinflammation, are tightly linked to their metabolic state. Emerging evidence suggests that metabolic reprogramming is a key driver of microglial activation, functional transitions, and interactions with neurons and other glial cells. This review summarizes current findings on the developmental origins, region-specific adaptations, and metabolic plasticity of microglia. We review lipid metabolism, energy utilization, and oxidative stress responses, which underlie immune regulation and neuroprotective functions. By integrating molecular, transcriptomic, and metabolomic insights, we provide a comprehensive understanding of microglial metabolism and highlight potential therapeutic strategies targeting metabolic pathways in neurodegenerative and central nervous system diseases.

## Introduction

Microglia, the resident immune cells of the central nervous system (CNS), play essential roles in neural development, homeostatic maintenance, and the pathogenesis of neurological disorders (Colonna and Butovsky, 2017). These cells are highly adaptable, which is closely tied to their metabolic state that determines whether they rely on oxidative phosphorylation (OXPHOS) or glycolysis, depending on context (Jung et al., 2025). When microglia are engaged in energy-demanding tasks such as phagocytosing debris or mounting inflammatory responses, they shift toward glycolysis to support rapid adenosine triphosphate (ATP) generation and effector functions (Bernier et al., 2020; Lepiarz-Raba et al., 2023; Sangineto et al., 2023). Conversely, under homeostatic conditions, microglia preferentially utilize mitochondrial OXPHOS to sustain long-term surveillance (Lauro and Limatola, 2020). This metabolic flexibility is critical for their immune competence and contributes to roles in both brain development and neurodegeneration (Colonna and Butovsky, 2017; Sadeghdoust et al., 2024a; Jung et al., 2025).

The impact of metabolic reprogramming is especially clear when immune signals stimulate microglia. In such cases, they switch from mitochondrial energy production to a quicker but less efficient glycolytic pathway. This shift is critical for enabling inflammation and is commonly seen in neurodegenerative diseases such as Alzheimer’s disease (AD) (Lauro and Limatola, 2020; Cheng et al., 2021). In AD, exposure to amyloidβ (Aβ) triggers this metabolic reprogramming via the mammalian target of rapamycin (mTOR)–hypoxia-inducible factor 1α (HIF1α) pathway, initially enhancing glycolysis to support phagocytosis, but chronic activation leads to metabolic collapse and microglial dysfunction, accelerating disease progression (Baik et al., 2019; Jung et al., 2025). Understanding how human microglia behave metabolically at different stages and activity levels is key to uncovering the complexities of brain immune responses and disease mechanisms. These insights could help identify new therapeutic targets to prevent or reverse the effects of dysfunctional microglia.

This review provides a comprehensive overview of microglial metabolism, from fetal development to aging. We examine how microglial metabolic states change across life stages, including fetal, neonatal, adolescent, adult, and elderly, and explore their various active roles: from maintaining normal function to responding to injury and disease. We also compare human and mouse microglia through gene expression, metabolic, and proteomic data to gain a more complete understanding. Additionally, we highlight the value of human stem cell-derived models and transplantation studies for investigating these dynamics, emphasizing in particular how the brain environment shapes microglial metabolism under both health and disease conditions. Finally, we outline future research directions and key technical challenges that need to be addressed to advance the field.

## Search Strategy

This manuscript provides a comprehensive review of major scientific databases, including PubMed, Scopus, and Web of Science. Peer-reviewed original and review articles addressing the metabolic reprogramming of microglia and its effects on development, disease, and therapeutic potential were included. The literature search employed the following key terms: metabolic reprogramming, microglia, functional states, lipid metabolism, energy metabolism, neuroinflammation, neurodegenerative disease, and human induced pluripotent stem cells. No restrictions were applied to the publication year.

## Changes in Metabolic State during the Developmental Stages of Human Microglia

### Microglia in the human fetal brain stage

In early development, microglia begin to enter the human brain between gestation weeks 5 and 6 (Monier et al., 2007; Menassa and Gomez-Nicola, 2018; Menassa et al., 2022), a critical period during which microglia proliferate and distribute throughout the brain (Kracht et al., 2020; Han et al., 2023). Mouse studies show that by embryonic day 14.5, important glycolysis genes, including lactate dehydrogenase A and triosephosphate isomerase 1, are highly expressed in microglia, indicating a high need for energy to support cell division (Hammond et al., 2019). This metabolic shift is crucial for their rapid expansion during early brain growth.

Genes important in phagocytic clearance of apoptotic cells, such as CD36 (a scavenger receptor) and triggering receptor expressed on myeloid cells 2 (TREM2), are also highly expressed in microglia at this stage (Hsieh et al., 2009; Yaqubi et al., 2023). This cleanup role is vital since cell turnover is extremely high in the fetal brain (Hattori, 2023). Equally importantly, at this stage, fetal microglia are active in lipid metabolism and lysosomal functions, facilitating breakdown and recycling debris (Prakash et al., 2025). Their metabolism is designed to support both phagocytosis and rapid proliferation, with glycolysis providing the energy and materials for building new cells (Prakash et al., 2025). Altogether, the well-tuned metabolic programming of microglia ensures proper development and early maintenance of the brain and may hold clues to understanding developmental disorders linked to microglial dysfunction.

### Metabolic state of microglia changes in response to environmental stimuli after birth

During early postnatal development, microglia undergo significant metabolic and functional maturation shaped by the brain environment. These changes enable critical processes such as synaptic pruning in infants and young children. At this stage, microglia strongly express genes related to synaptic pruning via complement-dependent pathways, such as C1q, C3, CR3, and CX3CR1, which mediate the removal of excess neural connections, a process essential for functional circuit formation, learning, and memory (Kracht et al., 2020; Cornell et al., 2022). Moreover, metabolic reprogramming during postnatal stages supports this functional shift in action and pruning capacity (Cantando et al., 2024; Mastenbroek et al., 2024).

Metabolically, postnatal microglia exhibit a hybrid energy profile that balances OXPHOS and glycolysis. Core metabolic regulators such as PGC1A, NDUFS1, and COX5B maintain mitochondrial respiration, while glycolytic enzymes hexokinase 2 (HK2), 6-phosphofructo-2-kinase/fructose-2,6-bisphosphatase 3 (PFKFB3), and pyruvate kinase M2 (PKM2) sustain rapid ATP production when microglia respond to neuronal activity (Lauro and Limatola, 2020). Their highly active lysosomal–autophagy systems, regulated by transcription factor EB (TFEB), CTSD, and lysosomal-associated membrane protein 1 (LAMP1), enable efficient clearance of synaptic debris and maintain neural homeostasis, both of which are essential for proper circuit refinement (Quick et al., 2023). Compared to their fetal-stage counterparts, childhood microglia have a more integrated metabolic profile yet maintain responsiveness to environmental cues. Disruption of this flexibility may predispose to neurodevelopmental disorders, as emerging evidence links metabolic dysregulation in maturing microglia to conditions such as autism spectrum disorders (Cantando et al., 2024).

### Human microglia undergo significant functional and metabolic adjustments during adolescence

In adolescence, microglia undergo profound changes in both their roles and transcriptomic profiles, essential for brain maturation. Recent single-cell and developmental analyses reveal that microglia in the adolescent prefrontal cortex exhibit enhanced metabolic activity, corresponding to their dynamic involvement in synaptic remodeling and plasticity (Lee et al., 2022a; Menassa et al., 2022; Schalbetter et al., 2022). This aligns with the broader pattern of brain plasticity during adolescence, including circuit refinement, which underlies emotional and cognitive development.

To meet these demands, microglia increase glycolytic flux through the activation of PFKFB3, PKM2, and LDHA, providing rapid energy for responding to environmental stimuli and modifying synapses. Mitochondrial activity may also increase during this time, supporting constant monitoring and interaction with neurons. This coordinated dual-fuel metabolism ensures both energetic flexibility and redox balance. Recent transcriptomic analyses reveal that adolescent microglia upregulate metabolic and signaling regulators such as HIF1A, mTORC1, and AMP-activated protein kinase, reflecting a unique bioenergetic signature distinct from earlier developmental stages (Sabogal-Guaqueta et al., 2023). Moreover, microglia in adolescent brains are particularly responsive to growth factors such as IGF-1, which support synaptic pruning and neuronal survival after injury (Eyolfson et al., 2020). Hormonal changes during puberty, including spikes in growth hormone and insulin-like growth factor 1, are also thought to influence microglial metabolic preferences and immune responses (Tavares et al., 2024). Understanding these changes is important because adolescence is when many psychiatric and neurodevelopmental conditions first emerge.

### Metabolic homeostasis of human microglia in adult steady state

In adulthood, microglia play a central role in maintaining CNS homeostasis. Hence, microglia must keep a stable yet primed metabolic profile. Traditionally, resting microglia were considered to rely predominantly on OXPHOS, driven by mitochondrial components such as NDUFS1, COX5B, and ATP5F1A, which ensure high energy efficiency during low-intensity surveillance (Lauro and Limatola, 2020). However, accumulating evidence indicates that even homeostatic microglia display a flexible metabolic state: actively importing glucose via SLC2A1 (GLUT1) and engaging moderate glycolysis through HK2, PFKFB3, and PKM2 to rapidly generate ATP for dynamic motility and process extension (Bernier et al., 2020). This combination of steady OXPHOS and standby glycolysis provides both energy efficiency and readiness, allowing microglia to quickly switch into an active “emergency responder” mode when needed. Lysosomal and autophagic regulators such as TFEB, LAMP1, and CTSB further contribute to turnover and proteostasis, preserving microglial function under physiological conditions. Maintaining this balance is especially important: in older adults, microglia show elevated expression of stress and inflammatory genes, suggesting they remain metabolically primed (Sadeghdoust et al., 2024b). Critically, when this balance is disrupted (e.g., in AD), microglia shift more permanently toward glycolysis, which is associated with sustained inflammation and impaired clearance of amyloid plaques (Sturno et al., 2025). Understanding how adult microglia maintain metabolic flexibility is vital for developing interventions that preserve neurological health and prevent harmful reprogramming.

### Aging microglia exhibit reactivation and shifts in metabolic programs

**Figure 1:**
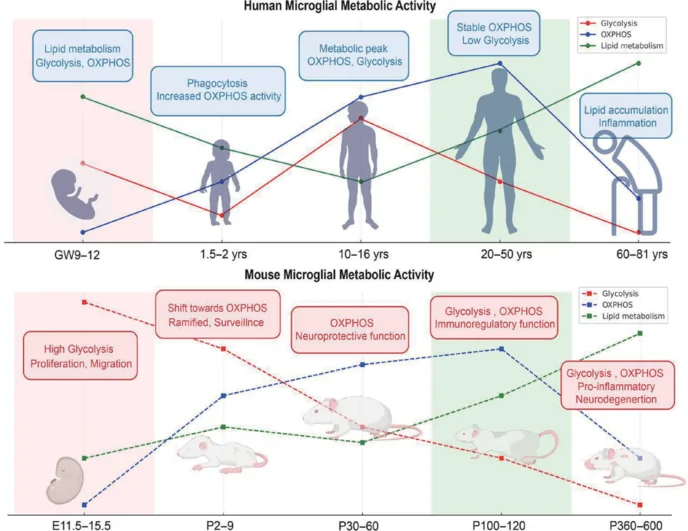
Developmental trajectories of microglial metabolism in humans and mice. Microglia undergo stage-specific metabolic remodeling across the lifespan. In humans (top), fetal microglia (GW9–12) rely on glycolysis and lipid metabolism, followed by enhanced OXPHOS activity during childhood (1.5–2 years) and a metabolic peak with balanced glycolysis and OXPHOS in adolescence (10–16 years). Adult microglia (20–50 years) maintain stable OXPHOS with reduced glycolysis, whereas aged microglia (60–81 years) exhibit lipid accumulation and inflammatory phenotypes. In mice (bottom), embryonic microglia (E11.5–15.5) display high glycolytic activity to support proliferation and migration, gradually transitioning to OXPHOS-dependent neuroprotection (P30–60) and immunoregulatory functions (P100–120). In aged mice (P360–600), microglia exhibit combined glycolytic and OXPHOS activity with pro-inflammatory signatures. Created with BioGDP (BioGDP.com). E: Embryonic day; GW: gestational week; OXPHOS: oxidative phosphorylation; P: postnatal day; yrs: years.

As the brain ages, increasing burdens of cellular debris and stress in microglia could drive the microglia state from homeostatic maintenance to reactive, inflammatory, and phagocytic metabolic states. In the aged brain, particularly within the white matter, microglia upregulate genes linked to phagocytosis and lipid metabolism, likely in response to myelin debris accumulation (Poliani et al., 2015; Olah et al., 2018). Interestingly, aged microglia from female mice exhibit elevated expression of disease-associated genes such as apolipoprotein E (*APOE*), *TREM2*, and lipoprotein lipase (*LPL*), along with increased glycolytic and OXPHOS activity, than male mice, possibly driven by post-menopausal estrogen decline (Safaiyan et al., 2021; Lynch, 2022; Li et al., 2023b). Another hallmark is the emergence of lipid-droplet-accumulating microglia, which are lipid-laden, phagocytosis-impaired, and pro-inflammatory (Marschallinger et al., 2020). In healthy aging, microglia in white matter often adopt the white matter-associated microglia phenotype, relying on TREM2 signaling and upregulating pathways for hypoxia response, glycolysis, cholesterol handling, and lysosomal activity, suggesting a protective adaptation (Safaiyan et al., 2021). However, in neurodegenerative disease contexts, microglia more often exhibit the disease-associated microglia (DAM) phenotype, characterized by sustained inflammation and reduced clearance of toxic aggregates. Ultimately, although aged microglia remain metabolically active, their adaptability is compromised, and they become overly dependent on specific energy pathways, leading to energy deficits and exacerbated neuroinflammation, which may drive age-related neurological decline. **Figure 1** and **Table 1** summarize dominant metabolic programs, associated functions, and representative markers in development and aging.

**Table 1:** Developmental and contextual shifts in microglial metabolism

Stage/context	Dominant metabolism	Key functions	Markers	Species differences	References
Fetal (GW5–20)	Glycolysis ↑	Rapid proliferation, apoptotic cell clearance, neurodevelopmental sculpting	LDHA, TPI1, PKM2, TREM2, and CD36	Human fetal microglia mature later and remain glycolytic longer, whereas vertebrate (mouse and zebrafish) microglia complete colonization earlier with faster oxidative transition	Matcovitch-Natan et al., 2016; Masuda et al., 2019
Postnatal	Balanced OXPHOS + glycolysis	Complement-mediated synaptic pruning, immune tolerance, developmental plasticity	C1q, C3, CX3CR1, and NF-κB	Human postnatal pruning/tolerance persists months beyond the brief, high-amplitude inflammatory waves seen in rodents	Geirsdottir et al., 2019; Hammond et al., 2019; Masuda et al., 2019
Adult homeostatic	OXPHOS + fatty acid oxidation (FAO)	Surveillance, motility, tissue homeostasis	P2RY12, SALL1, and TMEM119	Human microglia exhibit stable lipid-transport and anti-inflammatory modules; most vertebrates show higher turnover and lower TMEM119/SALL1 stability	Li and Barres, 2018; Hammond et al., 2019; Abels et al., 2021
Aged microglia	Glycolysis + lipid catabolism, mitochondrial decline	Chronic debris clearance, pro-inflammatory profile, senescence	APOE, LPL, LDHA, and oxidative stress genes	Aging human microglia exhibit stronger oxidative-stress and senescence genes (CDKN1A and GDF15) than rodent or fish counterparts	Masuda et al., 2019; Sharma et al., 2021; Prakash et al., 2025
White matter (myelin-rich)	Glycolysis + cholesterol catabolism	Myelin debris clearance, WAM response	CLEC7A, LPL, and APOE	Human white-matter microglia retain lipid stores and partial homeostatic traits, whereas rodent WAMs resolve rapidly	Keren-Shaul et al., 2017; Mancuso et al., 2019; Masuda et al., 2019
Diseased/neurodegenerative	Glycolytic shift + lysosomal/lipid programs	DAM-like state, amyloidβ clearance, inflammatory signaling	APOE, TREM2, LPL, and HIF-1α	Alzheimer’s disease associated microglia show attenuated lipid-droplet load but stronger IFN/lipid-axis activation than murine DAM	Keren-Shaul et al., 2017; Geirsdottir et al., 2019; Mancuso et al., 2019; Olah et al., 2020

Aβ: Amyloid-β; AD: Alzheimer’s disease; APOE: apolipoprotein E; C1q: complement component 1q; C3: complement component 3; CD36: cluster of differentiation 36; CX3CR1: CX3C chemokine receptor 1; FAO: fatty acid oxidation; GW: gestational week; HIF-1α: hypoxia-inducible factor 1-α; LDHA: lactate dehydrogenase A; LPL: lipoprotein lipase; NF-κB: nuclear factor κ-B; OXPHOS: oxidative phosphorylation; P2RY12: purinergic receptor P2Y12; PKM2: pyruvate kinase M2; SALL1: Sal-like protein 1; TMEM119: transmembrane protein 119; TPI1: triosephosphate isomerase 1; TREM2: triggering receptor expressed on myeloid cells 2.

## Metabolic Characteristics and Disease Associations of Different Functions/Activation States of Microglia

### Homeostatic microglia

In a healthy brain, microglia adopt a homeostatic state in which metabolism conserves energy while preserving readiness to respond rapidly. Homeostatic microglia typically exhibit low immune activity and rely mainly on OXPHOS and fatty acid β-oxidation, supported by mitochondrial proteins such as NDUFS1, COX5B, and ATP5F1A, to meet their energy demands, supporting their motile surveillance behavior (Afridi et al., 2020; Bielanin and Sun, 2023; Huang et al., 2024). Even in this resting condition, microglia show high glucose uptake through glucose transporter SLC2A1 and retain flexible glycolytic potential via HK2, PFKFB3, and PKM2, enabling rapid ATP generation for dynamic membrane remodeling and process extension (Bernier et al., 2020). When deprived of glucose, they maintain OXPHOS by engaging fatty acid oxidation through CPT1A and ACADM, thereby sustaining continuous morphological surveillance. Unlike many other immune cells that activate the Warburg effect, i.e., switching to glycolysis during activation, homeostatic microglia remain committed to OXPHOS, reflecting a “low-fuel, high-efficiency” energy model while retaining the ability to shift gears quickly if needed (Lauro and Limatola, 2020; Yang et al., 2021). This metabolic flexibility enables microglia to balance energy conservation with rapid responsiveness, a property essential for sustained brain surveillance. Understanding their baseline metabolic state may inform strategies to fine-tune microglial activity in neuroinflammatory diseases, where this essential balance is often disrupted.

### Injury-responsive microglia

When the brain experiences injury, ischemia, or other damage, microglia quickly transition into an injury-responsive state (interferon-responsive microglia [IRM]). Expression of genes involved in inflammation, chemotaxis, and phagocytosis, such as *CCL2*, interleukin-1 beta (*IL1B*), *CX3CR1*, and *TREM2*, is elevated in IRM, which moves microglia toward the injury site to clear damaged tissue (Hammond et al., 2019; Pallares-Moratalla and Bergers, 2024). To support this high-energy response, microglia switch from OXPHOS to glycolysis, coordinated by activation of *HIF1A* and *mTORC1*. Though glycolysis produces less energy per molecule of glucose, it is much faster and better suited for urgent needs. This “Warburg-like” effect enables microglia to upregulate glycolytic enzymes PFKFB3, PKM2, and LDHA to produce ATP quickly and sustain the burst of activity needed during injury response (Lauro and Limatola, 2020; Li et al., 2023a). In parallel, suppression of fatty-acid-oxidation genes such as *CPT1A* and *ACADM* redirects metabolic flux toward glycolysis. IRM microglia also upregulate genes related to lipid metabolism and lysosomal-clearance genes, including *LPL*, *ABCA1*, *CTSB*, and *LAMP1*, especially when cleaning up myelin debris and dead cells. This increased metabolic activity includes elevated mitochondrial stress and enhanced production of reactive oxygen species, which can further amplify inflammation if not properly regulated (Takeda et al., 2021; Sadeghdoust et al., 2024a). Typically, IRM is transient, reverting to a homeostatic profile once damage is resolved. In contrast, during chronic or unresolved injury, prolonged metabolic reprogramming can sustain microglia activation, thereby driving secondary neuroinflammation and exacerbating tissue damage.

### Disease-associated microglia

DAM represent a specialized activation state primarily observed in neurodegenerative diseases such as AD (Keren-Shaul et al., 2017). They exhibit a distinct gene signature and are characterized by the marked upregulation of TREM2, APOE, and lipid-processing enzymes, enabling them to detect and clear pathogenic aggregates, such as Aβ and tau (Liu et al., 2024; Jung et al., 2025). Metabolically, DAMs enhance glycolytic and lipid pathways to meet the energetic demands of phagocytosis and inflammation. Glycolytic enzymes such as HK2, PFKFB3, PKM2, and LDHA drive rapid ATP generation, while HIF1A and mTORC1 signaling maintain sustained glycolytic reprogramming. Concurrently, cholesterol-transport and lipid-metabolism proteins, including ABCA1, LPL, SOAT1, NPC2, and CYP46A1, regulate lipid uptake and trafficking, whereas lysosomal regulators such as TFEB, CTSD, and LAMP1 support degradation of aggregated proteins (Gao et al., 2023; Sadeghdoust et al., 2024a). Nonetheless, this metabolic reprogramming is often incomplete or dysregulated: DAMs struggle to clear toxic debris effectively and may instead perpetuate inflammation and neuronal stress (Sturno et al., 2025). Their dysregulated lipid handling, particularly cholesterol accumulation, further impairs their function over time. While the DAM phenotype initially serves as a defensive adaptation, its persistence and inefficiency contribute to disease progression. Targeting DAM-specific metabolic pathways, such as via TREM2 modulation, offers promising therapeutic avenues (Sun et al., 2025).

### White matter–associated microglia

White matter–associated microglia (WAM) represent a specialized microglial activation state that emerges predominantly in aging and demyelinating conditions. In aged mouse brains, especially within the white matter, WAM localize in nodular clusters. Genes involved in lipid clearance, cholesterol metabolism, and TREM2-dependent phagocytic pathways are upregulated in WAM, facilitating the removal of degenerated myelin (Safaiyan et al., 2021). WAM share certain gene signatures with DAM, including *Apoe* and *Clec7a*. However, they differ in that in normally aged mice, WAM can arise through an APOE-independent pathway, whereas in AD models, the development of microglia exhibiting DAM and WAM gene signatures depends on APOE function (Safaiyan et al., 2021). WAM upregulate lipid-processing and catabolic genes such as *LPL*, *FABP5*, *PLIN2*, *ABCA1*, and *CYP27A1*, which cooperate with lysosomal effectors LAMP1 and CTSB to support lipid degradation and debris turnover. Enhanced glycolytic activity, mediated by SLC2A1, HK2, and PFKFB3, and activation of hypoxia-response regulators such as HIF1A further sustain their energetic demands under white-matter stress (Ahn et al., 2022). Further analysis also shows WAM share DAM-like transcriptional profiles (e.g., *Apoe*, *B2m*, *Lyz2*, and *Clec7a*) and are characterized by enhanced lipid metabolism and phagocytic gene expression (Wei and Li, 2022).

### Proliferative region-associated microglia

Proliferative region-associated microglia (PAM) are a transient microglial subtype predominantly found in brain regions undergoing active development (e.g., the cortex and hippocampus), where high cellular turnover or neurogenesis occurs. These microglia exhibit increased proliferation and elevated phagocytic activity, aiding synaptic and neuronal remodeling. Transcriptomic profiling reveals upregulation of genes involved in cell cycle progression (Mki67 and Ccnb1), lysosomal function (CTSB and LAMP1), and lipid metabolism (Apoe, Lpl, and Fabp5), indicating robust energy generation and membrane biogenesis to support their remodeling capacity (Li et al., 2019). PAM also displays enhanced glycolytic and mitochondrial activity, with elevated expression of HK2, PFKFB3, and PKM2 promoting rapid ATP synthesis, complemented by PGC1A and NDUFS1 for mitochondrial oxidative function. Additionally, examination of microglial lipid and lipoprotein metabolism of PAM reveals upregulation of lipid uptake and metabolism genes such as *Apoe* and *Lpl*, reinforcing the role of PAM in metabolic adaptations for clearance and proliferation (Keren-Shaul et al., 2017; Loving and Bruce, 2020). Furthermore, during developmental apoptosis, microglia adopt states marked by increased lysosomal and lipid metabolism gene expression (e.g., TFEB, CTSD, and PLIN2), a remodeling axis that mirrors the functional requirements of PAM (Anderson et al., 2022). Notably, PAM may re-emerge in pathological conditions involving abnormal proliferation (e.g., gliomas or post-injury regeneration), suggesting shared metabolic underpinnings with their developmental roles.

### Other special states: Identification of more microglial subpopulations with technological advances

**Figure 2:**
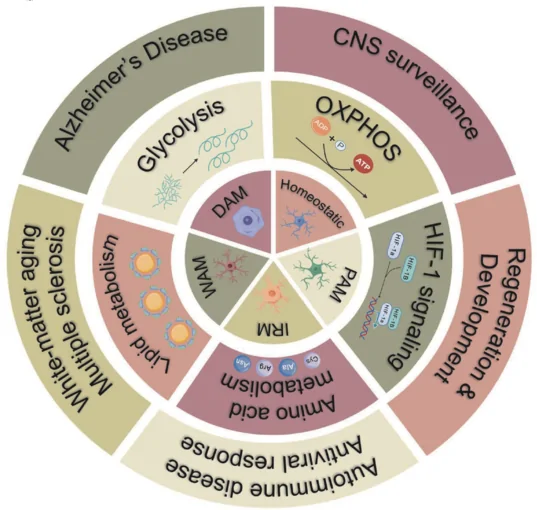
Integration of microglial states, metabolic pathways, and disease associations. A concentric network links core microglial states (center: homeostatic, DAM, WAM, IRM, and PAM) with dominant metabolic programs (middle ring: glycolysis, OXPHOS, lipid metabolism, amino acid metabolism, and HIF-1 signaling) and their corresponding physiological and pathological contexts (outer ring: CNS surveillance, Alzheimer’s disease, white matter aging, multiple sclerosis, autoimmunity, and regeneration). The diagram illustrates how state-specific metabolic rewiring underpins microglial functional diversity and contributes to disease pathogenesis. Created with BioGDP (BioGDP.com). AD: Alzheimer’s disease; DAM: disease-associated microglia; HIF-1: hypoxia-inducible factor-1; IRM: interferon-responsive microglia; OXPHOS: oxidative phosphorylation; PAM: proliferative-region–associated microglia; WAM: white-matter-associated microglia.

Recent advancements have identified microglial subtypes with distinct activation and metabolic profiles. Activated-response microglia express high levels of MHC-II and tissue repair genes (e.g., *Spp1*, *Gpnmb*, and *Dkk2*), and are enriched in AD risk genes, positioning them as a continuum between homeostatic microglia and DAM (Hammond et al., 2019; Antignano et al., 2023). These cells rely on increased glycolytic and lipid-metabolic activity, driven by PFKFB3, LDHA, and ACLY, to sustain energy for phagocytosis and extracellular-matrix remodeling. In contrast, late-response microglia, also referred to as IRM, arise in later disease stages under persistent type I interferon (IFN-I) signaling. They upregulate antiviral and immune-metabolic genes such as *ISG15*, *MX1*, *OAS1*, *HIF1A*, and *ACOD1*, promoting glycolysis and amino-acid catabolism to fuel inflammatory activation (Mathys et al., 2017; Hammond et al., 2019; Escoubas et al., 2024). Another subset, necroptotic microglia, engages inflammatory cell-death pathways mediated by RIPK1, RIPK3, and MLKL, releasing pro-inflammatory cytokines and altering metabolic flux toward reactive oxygen species-generating glycolysis (Huang et al., 2018; Yang et al., 2018). Collectively, these microglia subtypes illustrate a metabolic spectrum, from energy-efficient, homeostatic states heavily reliant on OXPHOS to glycolysis-driven, inflammation-oriented states. Such metabolic-functional coupling is central to microglial modulation of neuroinflammation and offers potential targets for treating both neurodegenerative and inflammatory brain disorders (Paolicelli et al., 2022; Jung et al., 2025). **Figure 2** and **Table 2** provide an integrative overview of microglial states, highlighting their metabolic signatures, potential mechanistic pathways, functional hallmarks, and therapeutic implications.

**Table 2:** Microglial states, metabolic signatures, mechanistic pathways, and therapeutic implications

Microglial state	Typical stimuli	Metabolic signature	Mechanistic pathways	Functional hallmarks	Therapeutic perspectives	Species differences	References
Homeostatic	–	OXPHOS / FAO	PPARγ–LXR and mitochondrial biogenesis	Surveillance, synaptic support, and trophic roles	Boost mitochondrial fitness (e.g., NAD+ boosters, and mitophagy enhancers)	Human homeostatic microglia show enriched lipid-handling and anti-inflammatory genes, while vertebrate (mouse and fish) microglia exhibit higher basal immune tone	Li and Barres, 2018; Hammond et al., 2019; Abels et al., 2021
IRM (interferon-responsive)	LPS, Poly(I:C), viral RNA	Glycolytic surge	Type I IFN–JAK–STAT, and mTOR–HIF1α axis	Chemotaxis, antiviral defense, and acute phagocytosis	Target IFN signaling or glycolysis modulators for acute inflammation	Human IRM show prolonged ISG expression and stronger antiviral tone; vertebrate IRM responses are transient	Matcovitch-Natan et al., 2016; Masuda et al., 2019
DAM (disease-associated)	Amyloid-β plaques, Tau aggregates, and oxidative stress	Glycolysis + lysosomal lipid metabolism	TREM2–APOE–mTOR, NF-κB, and lysosomal biogenesis	Plaque-proximal amyloid-β clearance, lipid droplet accumulation, and exhaustion risk	TREM2 agonists, APOE modulation, and cholesterol efflux enhancers	DAM core conserved, but human DAM displays weaker Cst7/Spp1 and stronger IFN–lipid modules	Keren-Shaul et al., 2017; Olah et al., 2020; Jorstad et al., 2023
WAM (white matter–associated)	Myelin debris, aging, and hypoxia	Glycolysis + cholesterol catabolism	LXR–SREBP and lipid droplet handling	Myelin debris clearance and white matter repair	Enhance lipid catabolism, and promote lysosomal acidification	Human WAMs persist longer in lesions with incomplete resolution; mouse/fish WAMs activate-deactivate rapidly	Mancuso et al., 2019; Masuda et al., 2019; Silva et al., 2021
PAM (proliferative region–associated)	Developmental neurogenesis signals	High glycolysis + nucleotide synthesis	c-Myc and mTORC1 signaling	Rapid proliferation and clonal expansion	mTOR inhibitors, and metabolic brakes to control proliferation	Human PAM-like clusters are rare (seen in tumors or development), whereas rodent and fish show broader proliferative re-activation	Keren-Shaul et al., 2017; Masuda et al., 2019; Silva et al., 2021
LDAM (lipid droplet-accumulating)	Oxidative stress, lipid overload, and amyloid exposure	Defective OXPHOS + lipid storage	Mitochondrial dysfunction, and reactive oxygen species accumulation	Pro-inflammatory, senescent-like phenotype	Antioxidants, lysosomal activators, and metabolic rebalancing	Lipid-droplet accumulation is conserved, but human LDAM show greater mitochondrial damage and persistent oxidative stress	Olah et al., 2020; Sharma et al., 2021; Jorstad et al., 2023; Prakash et al., 2025

Aβ: Amyloid-β; APOE: apolipoprotein E; CLEC7A: C-type lectin domain family 7 member A; c-Myc: cellular Myc; FAO: fatty acid oxidation; HIF-1α: hypoxia-inducible factor 1α; IFN: interferon; IRM: interferon-responsive microglia; ISG: interferon-stimulated gene; JAK: Janus kinase; LDAM: lipid droplet-accumulating microglia; LPL: lipoprotein lipase; LPS: lipopolysaccharide; LXR: liver X receptor; mTOR: mammalian/mechanistic target of rapamycin; mTORC1: mTOR complex 1; PAM: proliferative-region–associated microglia; Poly(I:C): polyinosinic–polycytidylic acid; PPARγ: peroxisome proliferator-activated receptor gamma; ROS: reactive oxygen species; SREBP: sterol regulatory element-binding protein; STAT: signal transducer and activator of transcription; TLR: Toll-like receptor; TREM2: triggering receptor expressed on myeloid cells 2; WAM: white-matter-associated microglia.

## Metabolic Differences between Human and Mouse Microglia

### Species-specific metabolic responses to inflammatory stimuli

Although human and mouse microglia share core functions such as surveillance and phagocytosis, they differ significantly in metabolic regulation and disease responses. For example, in response to inflammatory stimuli such as lipopolysaccharide (LPS), mouse and human microglia show species-specific features. Although both mouse and human induced pluripotent stem cell (iPSC)-derived microglia undergo a glycolytic shift upon Toll-like receptor 4 activation, their molecular mechanisms differ markedly. Mouse microglia enhance glycolysis primarily through upregulation of HK2, whereas human microglia rely on phosphofructokinase activity (PFKFB3) to sustain glycolytic flux. (Sabogal-Guaqueta et al., 2023). These findings align with broader transcriptomic comparisons showing that immune and metabolic genes are among the least conserved between mouse and human microglia (Gerrits et al., 2020). Together, these results underscore the necessity of considering species-specific metabolic wiring when interpreting microglial inflammatory responses, and they highlight the value of human stem cell–based systems, such as human microglia-containing brain organoids and transplantation-based chimeric brain model (Jiang et al., 2020), as powerful tools for advancing our understanding of human microglial metabolic states.

### Developmental timing and microglial metabolism across species

Single-cell analyses show that embryonic to early postnatal mouse microglia (E14.5 to P5) cluster more closely with human fetal-neonatal microglia, whereas juvenile-adult mouse microglia (P30 to P100) align better with human adolescent-adult stages, indicating a faster developmental timeline in mice compared to humans (Ginhoux and Prinz, 2015; Geirsdottir et al., 2019; Hammond et al., 2019; Masuda et al., 2019). Under inflammatory activation (e.g., LPS/Toll-like receptor 4), both species undergo a glycolytic shift, but mice predominantly upregulate HK2, while human microglia rely on phosphofructokinase/PFKFB3 activity, demonstrating species-specific glycolytic wiring (Sabogal-Guaqueta et al., 2023). In disease and aging contexts, human microglial states often show transcriptional and functional profiles absent or rare in mice, particularly in AD, where risk gene programs (e.g., *APOE*, *TREM2*, and *MHC-II*) are uniquely enriched in humans (Olah et al., 2020; Zhou et al., 2020; Mancuso et al., 2024). Notably, human iPSC-derived microglia transplanted into mouse brains retain human-specific transcriptional identity and functional heterogeneity, highlighting key species differences that persist even in chimeric environments (Hasselmann et al., 2019; Mancuso et al., 2019; Svoboda et al., 2019; Xu et al., 2020). These findings underscore the need for human-relevant models (iPSC-derived microglia, organoids, and chimeric systems) when analyzing microglial developmental and metabolic programs (**Figure 1**).

### Cross-species metabolic differences and implications for research

Species-specific differences are significant in metabolomic states and adaptability for microglia of different species. For instance, a study in mice confirms the essential role of CSF1R signaling (via CSF-1 and IL-34) in microglial survival, development, and maintenance (Easley-Neal et al., 2019), but direct comparative data in human microglia remain limited. Genetic factors also show distinct modulation of microglial behavior. For instance, APOE4 microglia in mouse AD models show elevated stress and metabolic dysregulation, yet the evolution of APOE in humans suggests that these effects may be more complex and not fully replicated in mice (Murphy et al., 2025). Regarding advanced microglial phenotypes, such as WAM observed in aged mouse white matter, their human counterparts have not been definitively identified. This highlights the need for caution in extrapolating mouse findings to human microglial biology (**Figure 1**).

### Unique metabolic environment of the human brain

The human brain operates in a unique metabolic environment, with high energy demands and sustained glucose supply. Microglia adapt dynamically, showing remarkable metabolic flexibility: when glucose is scarce, they can rapidly switch to glutaminolysis to maintain mitochondrial activity, surveillance, and debris clearance (Bernier et al., 2020). Microglia also express genes enabling the utilization of fatty acids and ketone bodies, suggesting adaptability in lipid-rich or fasting conditions (Lepiarz-Raba et al., 2023). Notably, the configuration of ketone body β-hydroxybutyrate can modulate microglial metabolism and attenuate Aβ-induced inflammation in human cells, spotlighting therapeutic potential (Jin et al., 2023). During inflammation, microglia undergo a Warburg-like metabolic shift, transitioning from oxidative phosphorylation to glycolysis to meet rapid energy and biosynthesis demands (Lauro and Limatola, 2020). However, species-specific differences in these metabolic responses remain understudied, especially regarding nutrient availability and fuel preference in human *vs*. mouse microglia. Addressing these knowledge gaps will advance our understanding of microglial function in health and disease and highlight the importance of investigating human-specific metabolic adaptations.

### Species differences in the transcriptional regulatory network of metabolism-related genes

At the genetic regulation level, microglial metabolism displays species-specific control. In both humans and mice, SALL1 is a critical lineage-determining transcription factor necessary for maintaining microglial identity. The loss of SALL1 leads to altered morphology and hyperinflammatory microglia (Holtman et al., 2017; Fixsen et al., 2023). The transcription factors PU.1, SALL1, and MAFB are established lineage-determining regulators sustaining microglial homeostasis. Deletion of *Mafb* in adult mouse microglia disrupts their identity and increases interferon and inflammation-related gene expression, underscoring its essential role in maintaining the homeostatic phenotype (Matcovitch-Natan et al., 2016). Developmental-stage-specific transcription factors are also notable: CEBPD peaks during puberty, regulating lipid metabolism pathways, while MAFB is highly expressed in pediatric microglia, and PRDM1 increases in adulthood, aligning with enhanced inflammatory gene signatures in microglia (Yaqubi et al., 2023). The transcriptional regulation of microglial metabolism differs substantially across nuclear receptor pathways. LXR/RXR signaling influences cholesterol homeostasis and immune function, supported by the effect of LXR on macrophage survival and innate responses (Joseph et al., 2004). Meanwhile, PPAR-δ plays a key role in limiting tissue damage during neuroinflammation, likely via metabolic regulation in microglia (Doroshenko et al., 2021). Recent reports indicate that nuclear receptor pathways, particularly PPAR-γ and LXR/RXR, play central roles in regulating microglial metabolism. PPAR-γ signaling has been shown to influence microglial energy utilization and anti-inflammatory responses, while LXR activation enhances cholesterol efflux and suppresses proinflammatory gene expression (Loving and Bruce, 2020; Yen and Yu, 2023; Zhang et al., 2024). These findings suggest that lipid-sensing pathways are critical modulators of microglial function.

## Metabolic Characteristics of Human Microglia *in vitro*/Induced Pluripotent Stem Cell Models and Xenotransplantation Models

### Induced pluripotent stem cell-derived microglial models

Recent studies have demonstrated that iPSC-derived microglia (iMGLs) exhibit a high degree of transcriptional similarity to their *in vivo* counterparts, expressing most of the key microglial genes essential for their function (Muffat et al., 2016; Abud et al., 2017; Douvaras et al., 2017; Pandya et al., 2017; Drager et al., 2022). Notably, when cultured with specific cytokines such as IL-34 combined with transforming growth factor-β1, iMGLs demonstrate partial restoration of native microglial features. However, *in vitro* iMGLs often lack the expression of critical microglial genes such as *SALL1* and *P2RY12*, all essential for maintaining microglial identity and function. This downregulation suggests that iMGLs may not fully reflect the true metabolic profile of microglia in the brain (Svoboda et al., 2019; Warden et al., 2023). Additionally, the *in vitro* environment does not replicate the complex interactions that occur within the brain, such as those with neurons and astrocytes. This lack of interaction can result in exaggerated inflammatory responses and metabolic activation in iMGLs, as shown by increased phagocytic activity and elevated levels of inflammatory cytokines when cultured alone (Stoberl et al., 2023).

While iMGLs serve as valuable tools for modeling human microglial metabolism, few studies have directly characterized their metabolic phenotype under varying nutrient or lipid environments. Data from microglial responses to lipid-rich substrates *in vivo* suggest that exposure to myelin debris triggers lipid metabolic programming, including upregulation of genes such as *ApoE* and *Fth1* (Hasel et al., 2023). The absence of such lipid cues in standard monoculture conditions may suggest why iMGLs could be skewed toward glycolysis, though this remains to be conclusively demonstrated (Dolan et al., 2023; Hasel et al., 2023). Similarly, iMGL transcriptomes often exhibit features reminiscent of early developmental microglia rather than mature adult forms, but a direct comparative analysis is still lacking. To address this gap, refining culture models, such as incorporating lipid-rich debris or building co-culture systems, is essential for aligning iMGL behavior more closely with *in vivo* adult microglia descriptions.

### Co-culture, brain organoid, and assembloid models

The iMGLs with human neurons, neural progenitors, or brain organoids better restore homeostatic and functional features compared with monoculture (Wu et al., 2025). In neuron-microglia co-culture, iMGLs downregulate pathogen-response pathways and exhibit a less pro-inflammatory cytokine profile at baseline and under LPS/IFNγ stimulation, indicating a shift toward surveillance-like states that more closely resemble primary microglia (Haenseler et al., 2017; Zhang et al., 2023). Within brain organoids, microglia integrate, extend processes, and promote neural network maturation, providing a 3D context to examine microglia-neuron crosstalk over development and disease (Abud et al., 2017; Ormel et al., 2018; Xu et al., 2021; Zhang et al., 2023). More recently, adhesion brain organoid systems achieved long-term survival and process ramifications of co-cultured human iPSC microglia and demonstrated neuroprotective effects, enabling extended analyses of function–metabolism coupling (Chen et al., 2025). Despite these advances, batch variability, integration time, and limited vascularization remain practical hurdles that can influence maturation and reproducibility (Zhang et al., 2023). Critically, the metabolic dimension in these co-culture and organoid systems is still under-explored; applying fluorescence lifetime imaging microscopy and related multiphoton approaches should allow real-time readouts of glycolysis/OXPHOS balance in microglia within 3D human contexts, closing a key knowledge gap (Barroso et al., 2023; Lacin and Yildirim, 2024). Finally, an organoid-xenotransplantation study underscores that a human brain-like environment is often required to fully reinstate adult-like microglial signatures and functions (Schafer et al., 2023).

### Human microglia chimeric brain models

To more accurately study human microglial biology, researchers have developed transplantation-based *in vivo* models in which mouse brains are engrafted with human iPSC-derived microglial progenitors, commonly termed xenografted microglial cells (Hasselmann et al., 2019; Mancuso et al., 2019; Svoboda et al., 2019; Xu et al., 2020; Jin et al., 2022a, b, 2024). In these chimeric systems, xenografted microglial cells populate and integrate into host neural networks, and, over several months, re-express signature human microglial markers such as *SALL1* and *P2RY12*, closely resembling adult human microglia post-transplantation (Hasselmann et al., 2019; Mancuso et al., 2019; Svoboda et al., 2019; Xu et al., 2020). Under demyelinating conditions (e.g., cuprizone), xenografted microglial cells mount responses analogous to resident microglia, upregulating genes such as *CD74* and *SPP1*, suggesting active participation in lipid metabolism and possibly glycolysis (Xu et al., 2020). Moreover, in AD models, xenografted microglia assemble around Aβ plaques and display hybrid transcriptional states that encompass both mouse DAM and human neurodegenerative microglia phenotypes, revealing their capacity for metabolic and functional reprogramming in pathology (McQuade et al., 2020; Mancuso et al., 2024). These findings strongly support the utility of chimeric models for exploring xenografted human microglial immunometabolism in health and disease. The capacity of human microglia to adapt to the brain microenvironment in these models reinforces their value for translational research into neurodegenerative conditions (Jin et al., 2022a, b, 2024; Warden et al., 2023; Papetti et al., 2025).

## Region-Specific Metabolic States and Functional Heterogeneity of Microglia

**Figure 3:**
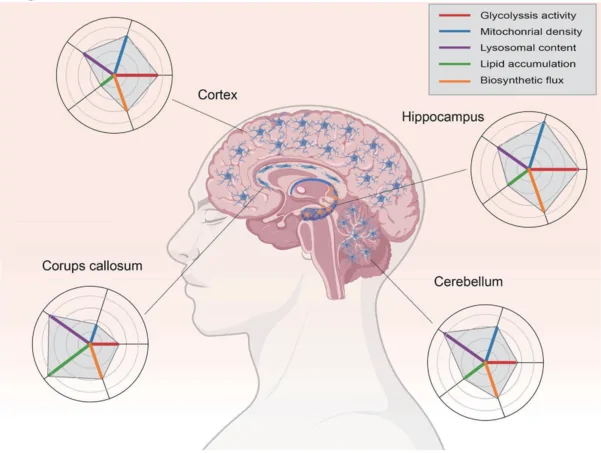
Regional heterogeneity of microglial metabolic programs in the human brain. Radar plots depict relative metabolic activities of microglia across cortical, hippocampal, cerebellar, and white matter (corpus callosum) regions. Parameters include glycolytic activity, mitochondrial density, lysosomal content, lipid accumulation, and biosynthetic flux. Distinct regional patterns emerge, with hippocampal microglia showing elevated glycolytic and biosynthetic activity, and corpus callosum microglia enriched for lipid accumulation and lysosomal content. These profiles highlight the spatial diversity of microglial metabolism across neuroanatomical domains. Created with BioGDP (BioGDP.com).

Microglia are not uniform throughout the brain; instead, they display region-specific phenotypes shaped by the local microenvironment. Comparative studies across regions such as the cortex, hippocampus, basal ganglia, and cerebellum have demonstrated differences in density, morphology, and expression of canonical microglial markers such as *CX3CR1* (De Biase et al., 2017; Tan et al., 2020). These differences are influenced by interactions with surrounding neurons and glial cells during development, which help microglia acquire a distinct regional identity (Li et al., 2019; Tan et al., 2020). Such specialization extends to metabolism as single-cell transcriptomic analyses reveal that microglia from different brain regions engage unique metabolic programs (Masuda et al., 2020; Hasel et al., 2023). For instance, cortical and hippocampal microglia show enhanced glycolytic signatures, whereas cerebellar and basal ganglia microglia exhibit distinct lipid and oxidative phosphorylation profiles (De Biase et al., 2017; Tan et al., 2020). This regional metabolic diversity is not merely a developmental by-product, but a functional adaptation that enables microglia to meet area-specific demands. Importantly, in neurodegenerative diseases such as AD, microglia undergo pronounced metabolic reprogramming, often shifting toward glycolysis and altered lipid handling to sustain inflammatory responses (Gao et al., 2023). Such changes, while initially protective, can exacerbate neuroinflammation and neuronal damage in vulnerable brain regions (Brandi et al., 2022; Lee et al., 2022b). Recognizing these region-specific metabolic patterns is therefore crucial for designing therapeutic strategies aimed at fine-tuning microglial activity in a spatially precise manner. **Figure 3** illustrates the regional heterogeneity of microglial metabolic profiles across major brain areas.

### Metabolic differences in microglia between white and gray matter regions

In white matter regions such as the corpus callosum, aging microglia transition into a distinct state termed white matter–associated microglia. These cells show enhanced lysosomal activity, cholesterol metabolism, and glycolysis, enabling them to clear myelin debris and accumulated lipids (Safaiyan et al., 2021; Ahn et al., 2022). This metabolic reprogramming is thought to preserve myelin integrity and axonal communication. By contrast, gray matter microglia in areas such as the cortex and hippocampus focus on synaptic support and neuronal plasticity. Under homeostatic conditions, microglia preferentially rely on OXPHOS, an efficient pathway that sustains their long-term surveillance role and synaptic monitoring (Gao et al., 2023). Transcriptomic analyses further reveal that gray matter microglia express genes related to neurotransmitter signaling and extracellular matrix interactions, reflecting their stable support of neuronal circuits (Brandi et al., 2022). With aging, gray matter microglia undergo comparatively subtle changes, such as modest upregulation of ion channels and matrix-related genes, but they do not form distinct clusters or dramatically alter their behavior as seen in WAM (Safaiyan et al., 2021; Brandi et al., 2022). Thus, white matter microglia exhibit greater metabolic flexibility and responsiveness, while gray matter microglia maintain a relatively stable, homeostatic profile. Understanding this regional dichotomy is critical for developing therapies for disorders such as AD, multiple sclerosis, or traumatic brain injury, where pathology may differentially affect white and gray matter microglia (Ahn et al., 2022; Gao et al., 2023).

### Distinctions in development and function of cortical and cerebellar microglia

Microglia in the cortex and cerebellum exhibit distinct developmental and functional specializations. Cerebellar microglia are highly active during synaptic remodeling, such as climbing fiber pruning, and show transcriptomic enrichment in immune and metabolic genes, including those related to glycolysis, antioxidant defense, and lipid handling (Grabert et al., 2016; De Biase et al., 2017). These features suggest enhanced resilience to metabolic stress and greater phagocytic capacity, supporting their role in motor learning and synaptic clearance (Nakayama et al., 2018; Guedes et al., 2023). In contrast, cortical microglia maintain a more stable, homeostatic phenotype, favoring OXPHOS and expressing genes associated with synaptic support and extracellular signaling (Safaiyan et al., 2021; Lepiarz-Raba et al., 2023). Overall, cortical microglia appear to adopt a more homogeneous, circuit-supportive role, while cerebellar microglia display greater recruit ability and reactivity during developmental and plasticity-associated contexts. This divergence likely reflects underlying metabolic programming but requires more refined region-specific analyses for confirmation.

### Hippocampus

The hippocampus, central to neurogenesis and memory, hosts microglia that appear relatively “active” even in adulthood. It has been proposed that these cells upregulate mitochondrial genes to sustain ongoing neuronal activity and the integration of newborn neurons. Transcriptomic studies indeed point to regional variation in microglial mitochondrial gene expression, though direct functional evidence is scarce (Grabert et al., 2016; De Biase et al., 2017). It remains speculative, for example, whether hippocampal microglia rely more on OXPHOS while sensory cortical microglia favor glycolysis, a hypothesis that requires validation with region-specific metabolic assays such as Seahorse flux analyses, isotope tracing, or fluorescence lifetime imaging microscopy imaging (Grabert et al., 2016). In disease contexts, stronger evidence emerges. In AD models, hippocampal microglia display increased glucose uptake, demonstrated by fluorodeoxyglucose-positron emission tomography and single-cell RNA sequencing, consistent with a glycolysis-driven, pro-inflammatory phenotype (Choi et al., 2021). More broadly, microglial metabolic reprogramming is tightly linked to polarization states, influencing neuroinflammation, synaptic remodeling, and cognition (Gao et al., 2023). Clarifying how hippocampal microglia adjust their metabolism could therefore reveal therapeutic opportunities to curb neuroinflammation and promote neurogenesis in neurodegenerative conditions.

### Basal ganglia

Microglia play a critical role in movement-related regions such as the striatum and substantia nigra, the primary targets in Parkinson’s disease. In Parkinson’s disease, the degeneration of dopaminergic neurons in the substantia nigra pars compacta triggers microglial activation, which can either exacerbate or mitigate neurodegeneration (Subramaniam and Federoff, 2017). Notably, microglia in these regions may upregulate the pentose phosphate pathway, specifically via increased glucose-6-phosphate dehydrogenase activity, to bolster NADPH production and counter oxidative stress. Dysregulation of the pentose phosphate pathway in microglia has been shown to intensify neuroinflammation and promote chronic dopaminergic neurodegeneration, leading to locomotor deficits (Tu et al., 2019). More broadly, chronic neurodegenerative conditions shift microglial metabolism from oxidative phosphorylation toward glycolysis, increasing glucose uptake, lactate production, and inflammatory signaling, while impairing phagocytic functions (Miao et al., 2023). These shifts suggest that microglia adopt a double-edged role in Parkinson’s disease, attempting to protect but potentially accelerating damage when metabolic control fails. Understanding these metabolic adaptations is crucial for identifying strategies that can shift microglial responses toward neuroprotection by targeting specific metabolic pathways.

### Mapping the metabolic landscape of region-specific microglia in the human brain

Mapping out how microglia use energy across different parts of the brain is becoming one of most exciting frontiers of neuroscience. Through advanced tools such as single-cell or single-nucleus RNA sequencing, spatial transcriptomics, and metabolic imaging, scientists are starting to delineate microglial metabolic behavior by region. The Human Microglia Atlas integrates nearly 90,000 human microglial cells across health and disease, revealing multiple transcriptionally distinct subpopulations that vary by region and pathology (Martins-Ferreira et al., 2025). Early evidence suggests that white matter microglia form a distinct WAM subtype, demonstrating unique gene expression and functional profiles, whereas gray matter microglia seem to lie along a more continuous metabolic spectrum (Safaiyan et al., 2021; Wang et al., 2025a). Spatial transcriptomics of aging mouse brain reveals pronounced inflammatory activation specifically in white matter tracts, underscoring regional vulnerability and metabolic response (Wang et al., 2025a). The next frontier lies in integrating transcriptomic and molecular metabolic data, such as gene modules for metabolism or in situ metabolite profiling, to pinpoint genuine regional metabolic subtypes. Spatial metabolomics and cross-regional single-cell multi-omics offer promise for mapping metabolic states in situ, enhancing our understanding of microglial adaptation, neuroinflammation, and protection in disorders such as AD or multiple sclerosis. Ultimately, identifying microglial metabolic signatures by region could enable precision therapies targeting both function and location.

## State-Specific Metabolic Features Revealed by Single-Cell Genomics and Metabolomics

Advanced technologies such as single-cell RNA sequencing, multi-omics, and metabolomics have revolutionized the study of microglia by revealing their remarkable metabolic flexibility and reprogramming in health and disease. Depending on environmental cues, including stress, injury, and neurodegeneration, microglia can shift between oxidative phosphorylation, glycolysis, and lipid metabolism. For example, in diabetic retinopathy, they adopt a glycolytic and purine metabolism profile that supports inflammatory activity (Lv et al., 2022), while multi-omic analyses in aging and AD have uncovered microglial subsets with enhanced lipid metabolism, particularly in response to Aβ accumulation (Olah et al., 2020; Wang et al., 2025b). Integrating metabolomic and transcriptomic data now allows researchers to trace how microglia “switch gears” to either drive inflammation or promote repair, highlighting new opportunities to therapeutically reprogram their metabolism toward protective functions.

### Single-cell transcriptomics reveals cellular states

Breakthroughs in single-cell RNA sequencing have revealed that microglia comprise dynamically regulated subpopulations marked by distinct metabolic profiles across development and disease. For example, a PAM subset, found in early postnatal white matter, displays elevated expression of oxidative phosphorylation and glycolytic genes such as *NDUFS1*, *COX5B*, *HK2*, *PFKFB3*, and *PKM2*, together with lipid-handling genes *Apoe*, *Lpl*, and *Fabp5*, reflecting a coordinated metabolic reprogramming during maturation (Li et al., 2019). In AD models such as 5×FAD mice, DAMs are characterized by upregulated lysosomal activity and glucose metabolism alongside suppressed homeostatic genes such as *P2ry12*, indicating a shift toward an inflammatory state (Olah et al., 2020; Liu et al., 2024). IRM, characterized by high expression of IRF7, IFI27, ISG15, and ACOD1, also displays enhanced glycolytic and amino-acid metabolic pathways during neuroinflammation (Escoubas et al., 2024). In aging white matter, WAM upregulate lipid metabolism and phagocytic pathways, including genes such as *LPL*, *PLIN2*, *ABCA1*, and *CYP27A1*, specializing in myelin debris clearance (Safaiyan et al., 2021; Ahn et al., 2022). These data underscore that microglial phenotypes are not fixed; they exist on a metabolic spectrum defined by state and location. Mapping these metabolic “fingerprints” via single-cell sequencing is paving the way for precision therapies that target specific microglial states to modulate disease progression, especially in AD.

### Multi-omics and spatial technologies reveal new layers of microglial metabolism

Recent advances in single-cell multi-omics and spatial omics are transforming our understanding of microglial metabolism and their inflammatory responses. A landmark study comparing mouse microglia with human iPSC-derived counterparts revealed that both shift from oxidative phosphorylation to glycolysis in response to LPS, but the molecular regulation differs: mouse microglia predominantly upregulate hexokinases (HK1 and HK2), while human counterparts elevate phosphofructokinases (PFKFB3 and PFK1) and downstream glycolytic enzymes PKM2 and LDHA, reflecting species-specific metabolic tuning (Sabogal-Guaqueta et al., 2023). In an AD context, microglia-specific deletion of PKM2, a key glycolytic enzyme, unexpectedly enhances both mitochondrial respiration and glycolytic capacity, flux through compensatory activation of *PGC1A*, *NDUFS1*, and *HIF1A*, promotes a DAM phenotype, and reduces amyloidbeta burden, suggesting that metabolic flexibility may be neuroprotective (Liu et al., 2024). Coupling single-nucleus RNA sequencing with spatial metabolomics further localizes these metabolic shifts, such as heightened activity of plaque-associated microglia, with elevated expression of *TREM2*, *APOE*, *TFEB*, and *ACOD1*, enabling precise mapping of metabolic microglial states (Mallach et al., 2024; Miyoshi et al., 2024; Wang et al., 2025a). Together, these multi-omics and spatial approaches uncover how microglial subpopulations differ functionally and metabolically, providing powerful insights for developing targeted therapies in neurodegenerative disorders.

### Unveiling microglial metabolism through large-scale metabolomics and proteomics

Recent innovations in proteomics and metabolomics have deepened our understanding of microglial metabolic reprogramming in AD. A large-scale proteomic study of AD brain tissue identified a microglial/glial metabolic module, via enzymes such as PKM2, LDHB, and GAPDH, whose expression correlates with Aβ load and is suppressed during acute inflammation (Johnson et al., 2020). Metabolomic profiling has further detected microglial metabolites such as itaconate, lactate, and cholesterol derivatives, consistent with the induction of *ACOD1* (also known as *IRG1*), *APOE*, and lipid-processing genes, such as *ABCA1*, *SOAT1*, and *LPL*, that integrate immunometabolism and lipid handling. In particular, DAM exhibit enhanced lysosomal and glucose-metabolic activity, characterized by *TFEB*, *CTSB*, and *LAMP1* upregulation, coupled with suppression of homeostatic markers P2RY12 and CX3CR1, reflecting activation of the TREM2–APOE regulatory axis (Krasemann et al., 2017). Integrative multi-omics approaches also demonstrate that microglial metabolism evolves in stages: during early AD, microglia increase glucose uptake and glycolysis, flux through PFKFB3, LDHA, and HIF1A, while later stages show mitochondrial dysfunction, lipid droplet accumulation (*PLIN2*, *CPT1A* dysregulation), and reduced clearance capacity—signs of metabolic exhaustion (Allen et al., 2019; Olah et al., 2020). These spatially resolved transcriptomic and metabolomic datasets further highlight regional differences, with plaque-associated microglia showing distinct activation and metabolic signatures (Olah et al., 2020). Together, these findings underscore that metabolic rewiring is central to microglial behavior. It is not merely a consequence of disease but a driver of how microglia adapt and function. Understanding these shifts provides promising therapeutic opportunities to restore microglial balance and attenuate neurodegeneration (Orihuela et al., 2016).

## Conclusion and Future Directions

**Figure 4:**
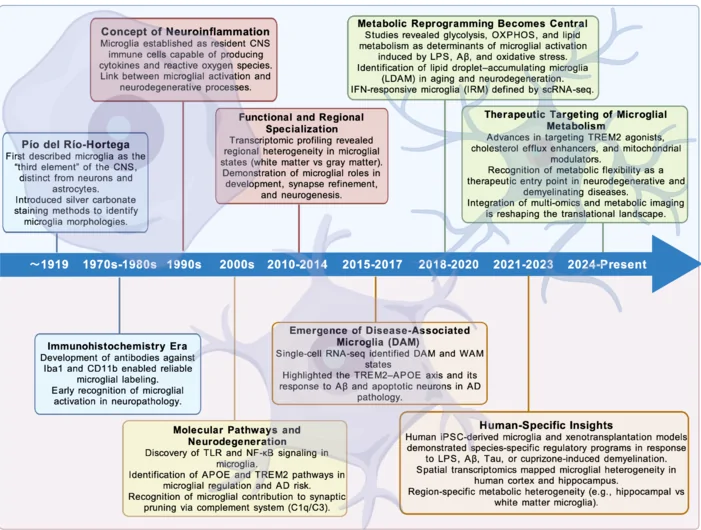
Timeline highlighting conceptual and methodological advances in microglia research. Timeline summarizing major conceptual and methodological advances in the study of microglia. Early work by Río-Hortega (~1919) (Iglesias-Rozas and Garrosa, 2012) established microglia as the “third element” of the CNS. In the 1970s–1980s, immunohistochemistry enabled selective identification of microglia (Iba1 and CD11b) (Hickey and Kimura, 1988; Ito et al., 1998), linking activation to neuropathology. The 1990s introduced the concept of neuroinflammation, recognizing microglia as cytokine-producing resident immune cells associated with neurodegeneration (McGeer and McGeer, 1995; Kreutzberg, 1996). In the 2000s, molecular pathways were identified, including TLR/NF-κB signaling, the APOE/TREM2 risk axis, and complement-mediated synaptic pruning (McGowan, 2006; Stevens et al., 2007; Guerreiro et al., 2013). Between 2010 and 2015 (Hickman et al., 2013; Heyer and Moore, 2016), transcriptomic profiling revealed functional and regional specialization of microglia across brain regions. In 2015–2017, single-cell RNA sequencing uncovered disease-associated microglia (DAM) (Keren-Shaul et al., 2017) and white-matter-associated microglia (WAM), underscoring the TREM2–APOE axis and its response to amyloidβ (Aβ) and apoptotic neurons in Alzheimer’s disease (Krasemann et al., 2017). From 2018 onwards, metabolic reprogramming emerged as a central determinant of microglial state transitions: LPS, Aβ, and oxidative stress were shown to drive shifts from oxidative phosphorylation to glycolysis, defining lipid droplet–accumulating microglia (LDAM) and interferon-responsive microglia (IRM) (Hasselmann et al., 2019; Mancuso et al., 2019; Masuda et al., 2019; Marschallinger et al., 2020). More recently (2020–2023), human iPSC-derived and xenotransplantation models revealed species-specific metabolic regulation in response to LPS, Aβ, Tau, or cuprizone-induced demyelination (Xu et al., 2020; Zhou et al., 2020; Jin et al., 2022b; Stoberl et al., 2023). Current efforts (2024–present) focus on therapeutic targeting of microglial metabolism, including TREM2 agonists, cholesterol efflux enhancers, and mitochondrial modulators, alongside integration of multi-omics and metabolic imaging for translational applications (Escoubas et al., 2024; Lloyd et al., 2024; Mancuso et al., 2024; Miyoshi et al., 2024; Sadeghdoust et al., 2024a; Martins-Ferreira et al., 2025; Papetti et al., 2025; Prakash et al., 2025; Wang et al., 2025a). Created with BioGDP (BioGDP.com). Aβ: Amyloid-β; AD: Alzheimer’s disease; APOE: apolipoprotein E; CNS: central nervous system; Iba1: ionized calcium-binding adapter molecule 1; IRM: interferon-responsive microglia; LDAM: lipid-droplet–accumulating microglia; MS: multiple sclerosis; NF-κB: nuclear factor kappa B; scRNA-seq: single-Cell RNA sequencing; TLR: Toll-like receptor; TREM2: triggering receptor expressed on myeloid cells 2; WAM: white-matter-associated microglia.

Microglia exhibit profound metabolic plasticity across developmental stages, adulthood, and aging, tightly coupling their metabolic states with functional transitions such as proliferation, surveillance, and disease-associated activation. Disruptions in this balance, exemplified by excessive glycolysis and lipid accumulation in AD, underscore the pathogenic consequences of metabolic dysregulation and highlight metabolism as a promising therapeutic target. **Figure 4** provides a historical overview of microglia research, highlighting milestones that frame current knowledge and guide future directions.

Advances in single-cell multi-omics, spatial profiling, and imaging technologies are now enabling a more precise mapping of human microglial metabolism. These tools reveal regional heterogeneity, disease-specific reprogramming, and potential epigenetic coupling mechanisms, deepening our understanding of how metabolites and signaling pathways such as HIF-1α, mTOR, and AMP-activated protein kinase drive phenotype transitions. Incorporating sex-specific and systemic metabolic factors will be crucial for developing personalized strategies.

Future progress will depend on the integration of *in vitro* models, patient-derived organoids, xenografts, and clinical imaging with multi-omics datasets analyzed through artificial intelligence. Building comprehensive human microglial atlases and designing microglia-specific metabolic probes will provide unprecedented insights. Ultimately, a refined understanding of microglial metabolic remodeling will not only advance basic neuroscience but also open new avenues for targeted interventions in neurodegenerative and neuroinflammatory diseases.

## Data Availability

*Not applicable.*

## References

[R1] Abels ER, Nieland L, Hickman S, Broekman MLD, El Khoury J, Maas SLN (2021). Comparative analysis identifies similarities between the human and murine microglial sensomes. Int J Mol Sci.

[R2] Abud EM (2017). iPSC-derived human microglia-like cells to study neurological diseases. Neuron.

[R3] Afridi R, Lee WH, Suk K (2020). Microglia gone awry: linking immunometabolism to neurodegeneration. Front Cell Neurosci.

[R4] Ahn K, Lee SJ, Mook-Jung I (2022). White matter-associated microglia: new players in brain aging and neurodegenerative diseases. Ageing Res Rev.

[R5] Allen BD, Apodaca LA, Syage AR, Markarian M, Baddour AAD, Minasyan H, Alikhani L, Lu C, West BL, Giedzinski E, Baulch JE, Acharya MM (2019). Attenuation of neuroinflammation reverses Adriamycin-induced cognitive impairments. Acta Neuropathol Commun.

[R6] Anderson SR, Roberts JM, Ghena N, Irvin EA, Schwakopf J, Cooperstein IB, Bosco A, Vetter ML (2022). Neuronal apoptosis drives remodeling states of microglia and shifts in survival pathway dependence. Elife.

[R7] Antignano I, Liu Y, Offermann N, Capasso M (2023). Aging microglia. Cell Mol Life Sci.

[R8] Baik SH, Kang S, Lee W, Choi H, Chung S, Kim JI, Mook-Jung I (2019). A breakdown in metabolic reprogramming causes microglia dysfunction in Alzheimer’s disease. Cell Metab.

[R9] Barroso M, Monaghan MG, Niesner R, Dmitriev RI (2023). Probing organoid metabolism using fluorescence lifetime imaging microscopy (FLIM): the next frontier of drug discovery and disease understanding. Adv Drug Deliv Rev.

[R10] Bernier LP, York EM, Kamyabi A, Choi HB, Weilinger NL, MacVicar BA (2020). Microglial metabolic flexibility supports immune surveillance of the brain parenchyma. Nat Commun.

[R11] Bielanin JP, Sun D (2023). Significance of microglial energy metabolism in maintaining brain homeostasis. Transl Stroke Res.

[R12] Brandi E, Torres-Garcia L, Svanbergsson A, Haikal C, Liu D, Li W, Li JY (2022). Brain region-specific microglial and astrocytic activation in response to systemic lipopolysaccharides exposure. Front Aging Neurosci.

[R13] Cantando I, Centofanti C, D’Alessandro G, Limatola C, Bezzi P (2024). Metabolic dynamics in astrocytes and microglia during post-natal development and their implications for autism spectrum disorders. Front Cell Neurosci.

[R14] Chen X, Sun G, Feng L, Tian E, Shi Y (2025). Human iPSC-derived microglial cells protect neurons from neurodegeneration in long-term cultured adhesion brain organoids. Commun Biol.

[R15] Cheng J, Zhang R, Xu Z, Ke Y, Sun R, Yang H, Zhang X, Zhen X, Zheng LT (2021). Early glycolytic reprogramming controls microglial inflammatory activation. J Neuroinflammation.

[R16] Choi H, Choi Y, Lee EJ, Kim H, Lee Y, Kwon S, Hwang DW, Lee DS, Alzheimer’s Disease Neuroimaging Initiative (2021). Hippocampal glucose uptake as a surrogate of metabolic change of microglia in Alzheimer’s disease. J Neuroinflammation.

[R17] Colonna M, Butovsky O (2017). Microglia function in the central nervous system during health and neurodegeneration. Annu Rev Immunol.

[R18] Cornell J, Salinas S, Huang HY, Zhou M (2022). Microglia regulation of synaptic plasticity and learning and memory. Neural Regen Res.

[R19] De Biase LM, Schuebel KE, Fusfeld ZH, Jair K, Hawes IA, Cimbro R, Zhang HY, Liu QR, Shen H, Xi ZX, Goldman D, Bonci A (2017). Local cues establish and maintain region-specific phenotypes of basal ganglia microglia. Neuron.

[R20] Dolan MJ (2023). Exposure of iPSC-derived human microglia to brain substrates enables the generation and manipulation of diverse transcriptional states in vitro. Nat Immunol.

[R21] Doroshenko ER, Drohomyrecky PC, Gower A, Whetstone H, Cahill LS, Ganguly M, Spring S, Yi TJ, Sled JG, Dunn SE (2021). Peroxisome proliferator-activated receptor-delta deficiency in microglia results in exacerbated axonal injury and tissue loss in experimental autoimmune encephalomyelitis. Front Immunol.

[R22] Douvaras P, Sun B, Wang M, Kruglikov I, Lallos G, Zimmer M, Terrenoire C, Zhang B, Gandy S, Schadt E, Freytes DO, Noggle S, Fossati V (2017). Directed differentiation of human pluripotent stem cells to microglia. Stem Cell Reports.

[R23] Drager NM, Sattler SM, Huang CT, Teter OM, Leng K, Hashemi SH, Hong J, Aviles G, Clelland CD, Zhan L, Udeochu JC, Kodama L, Singleton AB, Nalls MA, Ichida J, Ward ME, Faghri F, Gan L, Kampmann M (2022). A CRISPRi/a platform in human iPSC-derived microglia uncovers regulators of disease states. Nat Neurosci.

[R24] Easley-Neal C, Foreman O, Sharma N, Zarrin AA, Weimer RM (2019). CSF1R ligands IL-34 and CSF1 are differentially required for microglia development and maintenance in white and gray matter brain regions. Front Immunol.

[R25] Escoubas CC (2024). Type-I-interferon-responsive microglia shape cortical development and behavior. Cell.

[R26] Eyolfson E, Khan A, Mychasiuk R, Lohman AW (2020). Microglia dynamics in adolescent traumatic brain injury. J Neuroinflammation.

[R27] Fixsen BR (2023). SALL1 enforces microglia-specific DNA binding and function of SMADs to establish microglia identity. Nat Immunol.

[R28] Gao C, Jiang J, Tan Y, Chen S (2023). Microglia in neurodegenerative diseases: mechanism and potential therapeutic targets. Signal Transduct Target Ther.

[R29] Geirsdottir L (2019). Cross-species single-cell analysis reveals divergence of the primate microglia program. Cell.

[R30] Gerrits E, Heng Y, Boddeke E, Eggen BJL (2020). Transcriptional profiling of microglia; current state of the art and future perspectives. Glia.

[R31] Ginhoux F, Prinz M (2015). Origin of microglia: current concepts and past controversies. Cold Spring Harb Perspect Biol.

[R32] Grabert K, Michoel T, Karavolos MH, Clohisey S, Baillie JK, Stevens MP, Freeman TC, Summers KM, McColl BW (2016). Microglial brain region-dependent diversity and selective regional sensitivities to aging. Nat Neurosci.

[R33] Guedes JR, Ferreira PA, Costa J, Laranjo M, Pinto MJ, Reis T, Cardoso AM, Lebre C, Casquinha M, Gomes M, Shkatova V, Pereira M, Beltrao N, Hanuscheck N, Greenhalgh AD, Vogelaar CF, Carvalho AL, Zipp F, Cardoso AL, Peca J (2023). IL-4 shapes microglia-dependent pruning of the cerebellum during postnatal development. Neuron.

[R34] Guerreiro R (2013). TREM2 variants in Alzheimer’s disease. N Engl J Med.

[R35] Haenseler W, Sansom SN, Buchrieser J, Newey SE, Moore CS, Nicholls FJ, Chintawar S, Schnell C, Antel JP, Allen ND, Cader MZ, Wade-Martins R, James WS, Cowley SA (2017). A highly efficient human pluripotent stem cell microglia model displays a neuronal-co-culture-specific expression profile and inflammatory response. Stem Cell Reports.

[R36] Hammond TR, Dufort C, Dissing-Olesen L, Giera S, Young A, Wysoker A, Walker AJ, Gergits F, Segel M, Nemesh J, Marsh SE, Saunders A, Macosko E, Ginhoux F, Chen J, Franklin RJM, Piao X, McCarroll SA, Stevens B (2019). Single-cell RNA sequencing of microglia throughout the mouse lifespan and in the injured brain reveals complex cell-state changes. Immunity.

[R37] Han CZ (2023). Human microglia maturation is underpinned by specific gene regulatory networks. Immunity.

[R38] Hasel P, Aisenberg WH, Bennett FC, Liddelow SA (2023). Molecular and metabolic heterogeneity of astrocytes and microglia. Cell Metab.

[R39] Hasselmann J (2019). Development of a chimeric model to study and manipulate human microglia in vivo. Neuron.

[R40] Hattori Y (2023). The multifaceted roles of embryonic microglia in the developing brain. Front Cell Neurosci.

[R41] Heyer EE, Moore MJ (2016). Redefining the translational status of 80S monosomes. Cell.

[R42] Hickey WF, Kimura H (1988). Perivascular microglial cells of the cns are bone marrow-derived and present antigen in vivo. Science.

[R43] Hickman SE, Kingery ND, Ohsumi TK, Borowsky ML, Wang LC, Means TK, El Khoury J (2013). The microglial sensome revealed by direct RNA sequencing. Nat Neurosci.

[R44] Holtman IR, Skola D, Glass CK (2017). Transcriptional control of microglia phenotypes in health and disease. J Clin Invest.

[R45] Hsieh CL, Koike M, Spusta SC, Niemi EC, Yenari M, Nakamura MC, Seaman WE (2009). A role for TREM2 ligands in the phagocytosis of apoptotic neuronal cells by microglia. J Neurochem.

[R46] Huang Q, Wang Y, Chen S, Liang F (2024). Glycometabolic reprogramming of microglia in neurodegenerative diseases: insights from neuroinflammation. Aging Dis.

[R47] Huang Z, Zhou T, Sun X, Zheng Y, Cheng B, Li M, Liu X, He C (2018). Necroptosis in microglia contributes to neuroinflammation and retinal degeneration through TLR4 activation. Cell Death Differ.

[R48] Iglesias-Rozas J, Garrosa M (2012). The discovery of oligodendroglia cells by Río-Hortega: his original articles. Clin Neuropathol.

[R49] Ito D, Imai Y, Ohsawa K, Nakajima K, Fukuuchi Y, Kohsaka S (1998). Microglia-specific localisation of a novel calcium binding protein, Iba1. Brain Res Mol Brain Res.

[R50] Jiang P, Turkalj L, Xu R (2020). High-fidelity modeling of human microglia with pluripotent stem cells. Cell Stem Cell.

[R51] Jin LW, Di Lucente J, Ruiz Mendiola U, Suthprasertporn N, Tomilov A, Cortopassi G, Kim K, Ramsey JJ, Maezawa I (2023). The ketone body beta-hydroxybutyrate shifts microglial metabolism and suppresses amyloid-beta oligomer-induced inflammation in human microglia. FASEB J.

[R52] Jin M, Ma Z, Jiang P (2022). Generation of iPSC-based human-mouse microglial brain chimeras to study senescence of human microglia. STAR Protoc.

[R53] Jin M, Ma Z, Dang R, Zhang H, Kim R, Xue H, Pascual J, Finkbeiner S, Head E, Liu Y, Jiang P (2024). A trisomy 21-linked hematopoietic gene variant in microglia confers resilience in human iPSC models of Alzheimer’s disease. bioRxiv [preprint].

[R54] Jin M, Xu R, Wang L, Alam MM, Ma Z, Zhu S, Martini AC, Jadali A, Bernabucci M, Xie P, Kwan KY, Pang ZP, Head E, Liu Y, Hart RP, Jiang P (2022). Type-I-interferon signaling drives microglial dysfunction and senescence in human iPSC models of Down syndrome and Alzheimer’s disease. Cell Stem Cell.

[R55] Johnson ECB (2020). Large-scale proteomic analysis of Alzheimer’s disease brain and cerebrospinal fluid reveals early changes in energy metabolism associated with microglia and astrocyte activation. Nat Med.

[R56] Jorstad NL (2023). Comparative transcriptomics reveals human-specific cortical features. Science.

[R57] Joseph SB, Bradley MN, Castrillo A, Bruhn KW, Mak PA, Pei L, Hogenesch J, O’Connell R M, Cheng G, Saez E, Miller JF, Tontonoz P (2004). LXR-dependent gene expression is important for macrophage survival and the innate immune response. Cell.

[R58] Jung ES, Choi H, Mook-Jung I (2025). Decoding microglial immunometabolism: a new frontier in Alzheimer’s disease research. Mol Neurodegener.

[R59] Keren-Shaul H, Spinrad A, Weiner A, Matcovitch-Natan O, Dvir-Szternfeld R, Ulland TK, David E, Baruch K, Lara-Astaiso D, Toth B, Itzkovitz S, Colonna M, Schwartz M, Amit I (2017). A unique microglia type associated with restricting development of Alzheimer’s disease. Cell.

[R60] Kracht L, Borggrewe M, Eskandar S, Brouwer N, Chuva de Sousa Lopes SM, Laman JD, Scherjon SA, Prins JR, Kooistra SM, Eggen BJL (2020). Human fetal microglia acquire homeostatic immune-sensing properties early in development. Science.

[R61] Krasemann S (2017). The TREM2-APOE pathway drives the transcriptional phenotype of dysfunctional microglia in neurodegenerative diseases. Immunity.

[R62] Kreutzberg GW (1996). Microglia: a sensor for pathological events in the CNS. Trends Neurosci.

[R63] Lacin ME, Yildirim M (2024). Applications of multiphoton microscopy in imaging cerebral and retinal organoids. Front Neurosci.

[R64] Lauro C, Limatola C (2020). Metabolic reprograming of microglia in the regulation of the innate inflammatory response. Front Immunol.

[R65] Lee HL, Jung KM, Fotio Y, Squire E, Palese F, Lin L, Torrens A, Ahmed F, Mabou Tagne A, Ramirez J, Su S, Wong CR, Jung DH, Scarfone VM, Nguyen PU, Wood M, Green K, Piomelli D (2022). Frequent low-dose delta(9)-tetrahydrocannabinol in adolescence disrupts microglia homeostasis and disables responses to microbial infection and social stress in young adulthood. Biol Psychiatry.

[R66] Lee J, Kim SW, Kim KT (2022). Region-specific characteristics of astrocytes and microglia: a possible involvement in aging and diseases. Cells.

[R67] Lepiarz-Raba I, Gbadamosi I, Florea R, Paolicelli RC, Jawaid A (2023). Metabolic regulation of microglial phagocytosis: implications for Alzheimer’s disease therapeutics. Transl Neurodegener.

[R68] Li L, Cheng SQ, Sun YQ, Yu JB, Huang XX, Dong YF, Ji J, Zhang XY, Hu G, Sun XL (2023). Resolvin D1 reprograms energy metabolism to promote microglia to phagocytize neutrophils after ischemic stroke. Cell Rep.

[R69] Li Q, Barres BA (2018). Microglia and macrophages in brain homeostasis and disease. Nat Rev Immunol.

[R70] Li Q, Cheng Z, Zhou L, Darmanis S, Neff NF, Okamoto J, Gulati G, Bennett ML, Sun LO, Clarke LE, Marschallinger J, Yu G, Quake SR, Wyss-Coray T, Barres BA (2019). Developmental heterogeneity of microglia and brain myeloid cells revealed by deep single-cell RNA sequencing. Neuron.

[R71] Li X (2023). Transcriptional and epigenetic decoding of the microglial aging process. Nat Aging.

[R72] Liu Y, Kwok W, Yoon H, Ryu JC, Stevens P, Hawkinson TR, Shedlock CJ, Ribas RA, Medina T, Keohane SB, Scharre D, Bruschweiler-Li L, Bruschweiler R, Gaultier A, Obrietan K, Sun RC, Yoon SO (2024). Imbalance in glucose metabolism regulates the transition of microglia from homeostasis to disease-associated microglia stage 1. J Neurosci.

[R73] Lloyd AF, Martinez-Muriana A, Davis E, Daniels MJD, Hou P, Mancuso R, Brenes AJ, Sinclair LV, Geric I, Snellinx A, Craessaerts K, Theys T, Fiers M, De Strooper B, Howden AJM (2024). Deep proteomic analysis of microglia reveals fundamental biological differences between model systems. Cell Rep.

[R74] Loving BA, Bruce KD (2020). Lipid and lipoprotein metabolism in microglia. Front Physiol.

[R75] Lv K, Ying H, Hu G, Hu J, Jian Q, Zhang F (2022). Integrated multi-omics reveals the activated retinal microglia with intracellular metabolic reprogramming contributes to inflammation in STZ-induced early diabetic retinopathy. Front Immunol.

[R76] Lynch MA (2022). Exploring sex-related differences in microglia may be a game-changer in precision medicine. Front Aging Neurosci.

[R77] Mallach A, Zielonka M, van Lieshout V, An Y, Khoo JH, Vanheusden M, Chen WT, Moechars D, Arancibia-Carcamo IL, Fiers M, De Strooper B (2024). Microglia-astrocyte crosstalk in the amyloid plaque niche of an Alzheimer’s disease mouse model, as revealed by spatial transcriptomics. Cell Rep.

[R78] Mancuso R, Fattorelli N, Martinez-Muriana A, Davis E, Wolfs L, Van Den Daele J, Geric I, Premereur J, Polanco P, Bijnens B, Preman P, Serneels L, Poovathingal S, Balusu S, Verfaillie C, Fiers M, De Strooper B (2024). Xenografted human microglia display diverse transcriptomic states in response to Alzheimer’s disease-related amyloid-beta pathology. Nat Neurosci.

[R79] Mancuso R, Van Den Daele J, Fattorelli N, Wolfs L, Balusu S, Burton O, Liston A, Sierksma A, Fourne Y, Poovathingal S, Arranz-Mendiguren A, Sala Frigerio C, Claes C, Serneels L, Theys T, Perry VH, Verfaillie C, Fiers M, De Strooper B (2019). Stem-cell-derived human microglia transplanted in mouse brain to study human disease. Nat Neurosci.

[R80] Marschallinger J, Iram T, Zardeneta M, Lee SE, Lehallier B, Haney MS, Pluvinage JV, Mathur V, Hahn O, Morgens DW, Kim J, Tevini J, Felder TK, Wolinski H, Bertozzi CR, Bassik MC, Aigner L, Wyss-Coray T (2020). Lipid-droplet-accumulating microglia represent a dysfunctional and proinflammatory state in the aging brain. Nat Neurosci.

[R81] Martins-Ferreira R, Calafell-Segura J, Leal B, Rodriguez-Ubreva J, Martinez-Saez E, Mereu E, Pinho ECP, Laguna A, Ballestar E (2025). The Human Microglia Atlas (HuMicA) unravels changes in disease-associated microglia subsets across neurodegenerative conditions. Nat Commun.

[R82] Mastenbroek LJM, Kooistra SM, Eggen BJL, Prins JR (2024). The role of microglia in early neurodevelopment and the effects of maternal immune activation. Semin Immunopathol.

[R83] Masuda T, Sankowski R, Staszewski O, Bottcher C, Amann L, Sagar, Scheiwe C, Nessler S, Kunz P, van Loo G, Coenen VA, Reinacher PC, Michel A, Sure U, Gold R, Grun D, Priller J, Stadelmann C, Prinz M (2019). Spatial and temporal heterogeneity of mouse and human microglia at single-cell resolution. Nature.

[R84] Masuda T, Sankowski R, Staszewski O, Prinz M (2020). Microglia heterogeneity in the single-cell era. Cell Rep.

[R85] Matcovitch-Natan O (2016). Microglia development follows a stepwise program to regulate brain homeostasis. Science.

[R86] Mathys H, Adaikkan C, Gao F, Young JZ, Manet E, Hemberg M, De Jager PL, Ransohoff RM, Regev A, Tsai LH (2017). Temporal tracking of microglia activation in neurodegeneration at single-cell resolution. Cell Rep.

[R87] McGeer PL, McGeer EG (1995). The inflammatory response system of brain: implications for therapy of Alzheimer and other neurodegenerative diseases. Brain Res Brain Res Rev.

[R88] McGowan D (2006). Pruning processes. Nat Rev Neurosci.

[R89] McQuade A (2020). Gene expression and functional deficits underlie TREM2-knockout microglia responses in human models of Alzheimer’s disease. Nat Commun.

[R90] Menassa DA, Gomez-Nicola D (2018). Microglial dynamics during human brain development. Front Immunol.

[R91] Menassa DA, Muntslag TAO, Martin-Estebane M, Barry-Carroll L, Chapman MA, Adorjan I, Tyler T, Turnbull B, Rose-Zerilli MJJ, Nicoll JAR, Krsnik Z, Kostovic I, Gomez-Nicola D (2022). The spatiotemporal dynamics of microglia across the human lifespan. Dev Cell.

[R92] Miao J, Chen L, Pan X, Li L, Zhao B, Lan J (2023). Microglial metabolic reprogramming: emerging insights and therapeutic strategies in neurodegenerative diseases. Cell Mol Neurobiol.

[R93] Miyoshi E (2024). Spatial and single-nucleus transcriptomic analysis of genetic and sporadic forms of Alzheimer’s disease. Nat Genet.

[R94] Monier A, Adle-Biassette H, Delezoide AL, Evrard P, Gressens P, Verney C (2007). Entry and distribution of microglial cells in human embryonic and fetal cerebral cortex. J Neuropathol Exp Neurol.

[R95] Muffat J, Li Y, Yuan B, Mitalipova M, Omer A, Corcoran S, Bakiasi G, Tsai LH, Aubourg P, Ransohoff RM, Jaenisch R (2016). Efficient derivation of microglia-like cells from human pluripotent stem cells. Nat Med.

[R96] Murphy KB, Hu D, Wolfs L, Rohde SK, Fauro GL, Geric I, Mancuso R, De Strooper B, Marzi SJ (2025). The APOE isoforms differentially shape the transcriptomic and epigenomic landscapes of human microglia xenografted into a mouse model of Alzheimer’s disease. Nat Commun.

[R97] Nakayama H, Abe M, Morimoto C, Iida T, Okabe S, Sakimura K, Hashimoto K (2018). Microglia permit climbing fiber elimination by promoting GABAergic inhibition in the developing cerebellum. Nat Commun.

[R98] Olah M, Patrick E, Villani AC, Xu J, White CC, Ryan KJ, Piehowski P, Kapasi A, Nejad P, Cimpean M, Connor S, Yung CJ, Frangieh M, McHenry A, Elyaman W, Petyuk V, Schneider JA, Bennett DA, De Jager PL, Bradshaw EM (2018). A transcriptomic atlas of aged human microglia. Nat Commun.

[R99] Olah M (2020). Single cell RNA sequencing of human microglia uncovers a subset associated with Alzheimer’s disease. Nat Commun.

[R100] Orihuela R, McPherson CA, Harry GJ (2016). Microglial M1/M2 polarization and metabolic states. Br J Pharmacol.

[R101] Ormel PR, Vieira de Sa R, van Bodegraven EJ, Karst H, Harschnitz O, Sneeboer MAM, Johansen LE, van Dijk RE, Scheefhals N, Berdenis van Berlekom A, Ribes Martinez E, Kling S, MacGillavry HD, van den Berg LH, Kahn RS, Hol EM, de Witte LD, Pasterkamp RJ (2018). Microglia innately develop within cerebral organoids. Nat Commun.

[R102] Pallares-Moratalla C, Bergers G (2024). The ins and outs of microglial cells in brain health and disease. Front Immunol.

[R103] Pandya H, Shen MJ, Ichikawa DM, Sedlock AB, Choi Y, Johnson KR, Kim G, Brown MA, Elkahloun AG, Maric D, Sweeney CL, Gossa S, Malech HL, McGavern DB, Park JK (2017). Differentiation of human and murine induced pluripotent stem cells to microglia-like cells. Nat Neurosci.

[R104] Paolicelli RC (2022). Microglia states and nomenclature: A field at its crossroads. Neuron.

[R105] Papetti AV, Jin M, Ma Z, Stillitano AC, Jiang P (2025). Chimeric brain models: unlocking insights into human neural development, aging, diseases, and cell therapies. Neuron.

[R106] Poliani PL, Wang Y, Fontana E, Robinette ML, Yamanishi Y, Gilfillan S, Colonna M (2015). TREM2 sustains microglial expansion during aging and response to demyelination. J Clin Invest.

[R107] Prakash P, Randolph CE, Walker KA, Chopra G (2025). Lipids: emerging players of microglial biology. Glia.

[R108] Quick JD, Silva C, Wong JH, Lim KL, Reynolds R, Barron AM, Zeng J, Lo CH (2023). Lysosomal acidification dysfunction in microglia: an emerging pathogenic mechanism of neuroinflammation and neurodegeneration. J Neuroinflammation.

[R109] Sabogal-Guaqueta AM, Marmolejo-Garza A, Trombetta-Lima M, Oun A, Hunneman J, Chen T, Koistinaho J, Lehtonen S, Kortholt A, Wolters JC, Bakker BM, Eggen BJL, Boddeke E, Dolga A (2023). Species-specific metabolic reprogramming in human and mouse microglia during inflammatory pathway induction. Nat Commun.

[R110] Sadeghdoust M, Das A, Kaushik DK (2024). Fueling neurodegeneration: metabolic insights into microglia functions. J Neuroinflammation.

[R111] Sadeghdoust M, Das A, Kaushik DK (2024). Fueling neurodegeneration: metabolic insights into microglia functions. J Neuroinflammation.

[R112] Safaiyan S, Besson-Girard S, Kaya T, Cantuti-Castelvetri L, Liu L, Ji H, Schifferer M, Gouna G, Usifo F, Kannaiyan N, Fitzner D, Xiang X, Rossner MJ, Brendel M, Gokce O, Simons M (2021). White matter aging drives microglial diversity. Neuron.

[R113] Sangineto M, Ciarnelli M, Cassano T, Radesco A, Moola A, Bukke VN, Romano A, Villani R, Kanwal H, Capitanio N, Duda L, Avolio C, Serviddio G (2023). Metabolic reprogramming in inflammatory microglia indicates a potential way of targeting inflammation in Alzheimer’s disease. Redox Biol.

[R114] Schafer ST, Mansour AA, Schlachetzki JCM, Pena M, Ghassemzadeh S, Mitchell L, Mar A, Quang D, Stumpf S, Ortiz IS, Lana AJ, Baek C, Zaghal R, Glass CK, Nimmerjahn A, Gage FH (2023). An in vivo neuroimmune organoid model to study human microglia phenotypes. Cell.

[R115] Schalbetter SM, von Arx AS, Cruz-Ochoa N, Dawson K, Ivanov A, Mueller FS, Lin HY, Amport R, Mildenberger W, Mattei D, Beule D, Foldy C, Greter M, Notter T, Meyer U (2022). Adolescence is a sensitive period for prefrontal microglia to act on cognitive development. Sci Adv.

[R116] Sharma K, Bisht K, Eyo UB (2021). A comparative biology of microglia across species. Front Cell Dev Biol.

[R117] Silva NJ, Dorman LC, Vainchtein ID, Horneck NC, Molofsky AV (2021). In situ and transcriptomic identification of microglia in synapse-rich regions of the developing zebrafish brain. Nat Commun.

[R118] Stevens B, Allen NJ, Vazquez LE, Howell GR, Christopherson KS, Nouri N, Micheva KD, Mehalow AK, Huberman AD, Stafford B, Sher A, Litke AM, Lambris JD, Smith SJ, John SW, Barres BA (2007). The classical complement cascade mediates CNS synapse elimination. Cell.

[R119] Stoberl N, Maguire E, Salis E, Shaw B, Hall-Roberts H (2023). Human iPSC-derived glia models for the study of neuroinflammation. J Neuroinflammation.

[R120] Sturno AM, Hassell JE, Lanaspa MA, Bruce KD (2025). Do microglia metabolize fructose in Alzheimer’s disease?. J Neuroinflammation.

[R121] Subramaniam SR, Federoff HJ (2017). Targeting microglial activation states as a therapeutic avenue in Parkinson’s disease. Front Aging Neurosci.

[R122] Sun Y, Wei K, Liao X, Wang J, Gao L, Pang B (2025). Lipid metabolism in microglia: Emerging mechanisms and therapeutic opportunities for neurodegenerative diseases (Review). Int J Mol Med.

[R123] Svoboda DS, Barrasa MI, Shu J, Rietjens R, Zhang S, Mitalipova M, Berube P, Fu D, Shultz LD, Bell GW, Jaenisch R (2019). Human iPSC-derived microglia assume a primary microglia-like state after transplantation into the neonatal mouse brain. Proc Natl Acad Sci U S A.

[R124] Takeda H, Yamaguchi T, Yano H, Tanaka J (2021). Microglial metabolic disturbances and neuroinflammation in cerebral infarction. J Pharmacol Sci.

[R125] Tan YL, Yuan Y, Tian L (2020). Microglial regional heterogeneity and its role in the brain. Mol Psychiatry.

[R126] Tavares MR, Wasinski F, Metzger M, Donato J (2024). Impact of growth hormone on microglial and astrocytic function. J Integr Neurosci.

[R127] Tu D, Gao Y, Yang R, Guan T, Hong JS, Gao HM (2019). The pentose phosphate pathway regulates chronic neuroinflammation and dopaminergic neurodegeneration. J Neuroinflammation.

[R128] Wang L (2025). Spatial transcriptomics of the aging mouse brain reveals origins of inflammation in the white matter. Nat Commun.

[R129] Wang Z, Zhang L, Qin C (2025). Alzheimer’s disease pathogenesis: standing at the crossroad of lipid metabolism and immune response. Mol Neurodegener.

[R130] Warden AS, Han C, Hansen E, Trescott S, Nguyen C, Kim R, Schafer D, Johnson A, Wright M, Ramirez G, Lopez-Sanchez M, Coufal NG (2023). Tools for studying human microglia: In vitro and in vivo strategies. Brain Behav Immun.

[R131] Wei Y, Li X (2022). Different phenotypes of microglia in animal models of Alzheimer disease. Immun Ageing.

[R132] Wu J, Chen X, Zhang J, Wettschurack K, Robinson M, Li W, Zhao Y, Yoo YE, Deming BA, Abeyaratna AD, Que Z, Du D, Tegtmeyer M, Yuan C, Skarnes WC, Rochet JC, Wu LJ, Yang Y (2025). Human microglia in brain assembloids display region-specific diversity and respond to hyperexcitable neurons carrying SCN2A mutation: microglial diversity and response in assembloids. bioRxiv [Preprint].

[R133] Xu R, Li X, Boreland AJ, Posyton A, Kwan K, Hart RP, Jiang P (2020). Human iPSC-derived mature microglia retain their identity and functionally integrate in the chimeric mouse brain. Nat Commun.

[R134] Xu R, Boreland AJ, Li X, Erickson C, Jin M, Atkins C, Pang ZP, Daniels BP, Jiang P (2021). Developing human pluripotent stem cell-based cerebral organoids with a controllable microglia ratio for modeling brain development and pathology. Stem Cell Reports.

[R135] Yang J, Zhao Y, Zhang L, Fan H, Qi C, Zhang K, Liu X, Fei L, Chen S, Wang M, Kuang F, Wang Y, Wu S (2018). RIPK3/MLKL-mediated neuronal necroptosis modulates the M1/M2 polarization of microglia/macrophages in the ischemic cortex. Cereb Cortex.

[R136] Yang S, Qin C, Hu ZW, Zhou LQ, Yu HH, Chen M, Bosco DB, Wang W, Wu LJ, Tian DS (2021). Microglia reprogram metabolic profiles for phenotype and function changes in central nervous system. Neurobiol Dis.

[R137] Yaqubi M, Groh AMR, Dorion MF, Afanasiev E, Luo JXX, Hashemi H, Sinha S, Kieran NW, Blain M, Cui QL, Biernaskie J, Srour M, Dudley R, Hall JA, Sonnen JA, Arbour N, Prat A, Stratton JA, Antel J, Healy LM (2023). Analysis of the microglia transcriptome across the human lifespan using single cell RNA sequencing. J Neuroinflammation.

[R138] Yen JJ, Yu (2023). The role of ApoE-mediated microglial lipid metabolism in brain aging and disease. Immunometabolism (Cobham).

[R139] Zhang R, Wuerch E, Yong VW, Xue M (2024). LXR agonism for CNS diseases: promises and challenges. J Neuroinflammation.

[R140] Zhang W, Jiang J, Xu Z, Yan H, Tang B, Liu C, Chen C, Meng Q (2023). Microglia-containing human brain organoids for the study of brain development and pathology. Mol Psychiatry.

[R141] Zhou Y (2020). Human and mouse single-nucleus transcriptomics reveal TREM2-dependent and TREM2-independent cellular responses in Alzheimer’s disease. Nat Med.

